# A measurement study of the relationship between personality traits and learning emotions among music learners: a psychometric analysis in an educational context

**DOI:** 10.3389/fpsyg.2026.1810569

**Published:** 2026-04-30

**Authors:** Xuan Zhou, Shu Wen, Song Dong

**Affiliations:** 1 School of Music and Dance, Xihua University, Chengdu, Sichuan, China; 2 The Kyrgyz National University named after Zhusup Balasagyn, Bishkek, Kyrgyzstan; 3 Department of Global Convergence, Kangwon National University, Chuncheon, Republic of Korea

**Keywords:** Big Five personality traits, Chinese college students, learning emotions, music education, psychometric validation, structural equation modeling

## Abstract

This study examined the relationships between Big Five personality traits and learning emotions among Chinese undergraduate music students, while also validating psychometric instruments adapted to the music education context. A cross-sectional quantitative design was employed with a sample of 298 undergraduate music students drawn from three Chinese universities. The Chinese Big Five Personality Inventory-Short Form (CBF-PI-SF) served as the measure of personality traits, and an adapted version of the Achievement Emotions Questionnaire-Short (AEQ-S) was used to assess learning emotions in the context of music learning. Confirmatory factor analysis demonstrated adequate factorial validity for both instruments, with most fit indices meeting recommended thresholds and the overall pattern supporting acceptable model fit. Structural equation modeling revealed that personality traits collectively accounted for substantial variance in positive (R^2^ = 0.42) and negative emotions (R^2^ = 0.38) as well as boredom (R^2^ = 0.29). Extraversion (*b* = 0.35, *p* < 0.001) and openness (b = 0.28, *p* < 0.001) proved to be the strongest predictors of positive learning emotions, and neuroticism emerged as the strongest predictor of negative emotions (*b* = 0.42, *p* < 0.001). Openness showed a strong negative association with boredom (*b* = −0.32, *p* < 0.001). Agreeableness did not have significant relationships with any emotional outcome. These findings extend the application of Control-Value Theory in music education and also provide empirical support for the use of personality-based teaching methods. The instruments examined, owing to their validity, can serve as useful tools in conducting subsequent studies to investigate individual differences in emotional experiences in the context of creative and performance-based education.

## 1 Introduction

The intersection of personality psychology and educational science has gained considerable attention in contemporary research, particularly in discipline-specific contexts such as music education (Price, 2026). Learning music is a distinctive form of education in which learning and pedagogical processes are difficult to fully explain solely through cognitive, emotional, and behavioral perspectives (Amianto et al., 2026); therefore, it provides an ideal context for examining the complex relationships between individual differences in personality traits and emotional experiences in the learning process (Carini et al., 2025). Recent psychometric advances have improved measurement precision in personality and emotion research, enabling more accurate estimation of personality constructs and affective responses (Rivieccio et al., 2025). These advances have opened new avenues for understanding how fundamental aspects of personality shape emotional experiences in learning processes, particularly in performance and creative contexts (Guerrero et al., 2025).

Personality traits, as defined by well-established models such as the Big Five, have been widely studied in relation to academic performance and learning outcomes across various disciplines (Rodríguez Hernández et al., 2025). However, the specific mechanisms through which personality traits influence learning emotions in music education remain relatively under-researched (Váradi, 2022). Learning emotions, referring to the affective states experienced during learning activities, play an important role in motivation, engagement, and ultimately learning outcomes (Rosenblad et al., 2025). These constructs present distinct measurement challenges, requiring robust psychometric instruments capable of capturing both stable personality traits and the dynamic nature of context-specific learning emotions (Zmork-Martínez et al., 2025). Recent studies have emphasized the need for validated assessment tools that can provide reliable and valid measurements of such complex psychological constructs (Glaveanu and Maier, 2025).

Research in music education psychology increasingly recognizes the importance of empirical studies that integrate personality psychology with educational research methodologies (Huang, 2025). Music learning differs from other subjects due to its performance, creativity, and emotional demands, including performance anxiety (Varela-Figueroa et al., 2025), creative expression (Husain et al., 2025), technical skill development (Kolenbrander and Michaels, 2025), and aesthetic appreciation (Bogunović, 2024). These characteristics highlight music education as a valuable domain for examining how individual differences in personality relate to specific learning emotions and behavioral performance (Leite et al., 2025). In addition, the development and validation of psychometric instruments tailored to music education contexts represent a crucial area of research that can advance both theoretical understanding and educational practice (Özalp et al., 2025).

Yet, despite the fact that the importance of personality traits and learning emotions in the teaching process is being increasingly recognized (Mollaioli et al., 2025), the nature of the relationships between these constructs is not well understood in the context of music learning (Gaspar-Pérez et al., 2025). Although previous studies have confirmed the existence of general relationships between personality variables and academic performance (Santillo et al., 2025), the finer details of how specific personality variables interact with the emotions of music learners have not been subjected to thorough psychometric scrutiny (Lin et al., 2024). This knowledge gap is especially problematic considering the unique demands of music education, which require both cognitive processing and emotional engagement in ways that are differentially sensitive to personality traits (Perrotta and Eleuteri, 2024). The lack of measurement tools and research specifically designed to analyze these relationships limits our ability to develop evidence-based approaches to optimize music education practices and address the needs of diverse learners in music-related contexts (Fine, 2024).

The main aims of this research are as follows:

To determine the applicability and psychometric properties of validated personality trait measures and learning emotion scales in music education settings.
To examine the associations between personality trait dimensions and types of learning emotions among music students using quantitative analysis.
To identify practical implications of personality–emotion interactions for music education and propose directions for future research.

The study addresses a key gap in personality and music education research, as it provides a psychometric analysis of personality–emotion interactions during music learning (Chen Y. et al., 2024). The results will be valuable in terms of theoretical contributions to understanding the role of individual differences in educational experiences within creative domains by extending existing personality–learning frameworks to music education (Boroń et al., 2024). Practically, the research will equip music teachers, curriculum designers, and educational psychologists with evidence-based resources and strategies to personalize instruction and meet the needs of diverse learners (Penado Abilleira et al., 2024). The validated psychometric instruments developed as part of the current study will serve as valuable resources for future research in music education psychology and may lead to more efficient assessment procedures and interventions (Vispoel et al., 2024). Furthermore, the study’s methodology and findings can guide related research in other creative and performance-based learning domains, contributing to a broader understanding of personality–emotion relationships in specialized educational contexts (Recław et al., 2024).

The primary contributions of this work include the adaptation and psychometric validation of existing instruments, specifically the CBF-PI-SF and the AEQ-S, for application in the music education context, the empirical identification of key relationships between personality trait dimensions and specific categories of learning emotions in music learners, and the establishment of a statistical modeling framework applicable to similar studies in other learning domains. In addition, the study provides evidence-based recommendations for personalized music education practices based on individual personality traits and extends existing personality–learning models to creative and performance-based educational settings (Bieleke et al., 2021).

The rest of this paper is structured as follows. Section 2 presents a literature review of studies on personality traits, learning emotions, and their interrelationships in education, with a focus on music education. Section 3 describes the methodology, including research design, sample, psychometric instrument development and validation, and statistical analysis methods. Section 4 reports the findings of psychometric validation, descriptive statistics, correlation analysis, and statistical modeling of personality–emotion relationships. Section 5 discusses the findings in relation to existing theory and research, outlines implications for music education practice, and identifies study limitations. Finally, Section 6 concludes the paper by summarizing the main findings and offering recommendations for researchers and practitioners in music education.

## 2 Literature review

This section synthesizes the theoretical and empirical foundations directly relevant to the relationship between Big Five personality traits and learning emotions in music education. The review is organized around four core areas: (1) personality psychology frameworks as applied to educational achievement and learning processes; (2) the Control-Value Theory of achievement emotions and its application to domain-specific learning contexts; (3) individual differences in music education psychology, with particular attention to emotional and motivational correlates; and (4) psychometric assessment and validation methodology in educational research. Rather than providing a broad survey of personality psychology, the review is deliberately focused on literature most directly pertinent to the research questions: how personality traits predict learning emotion profiles in music students, and whether standardized instruments can be validly adapted for this specialized domain. A comparative synthesis of recent studies (2024–2026) highlights the methodological landscape, followed by identification of gaps that justify the present investigation.

### 2.1 Theoretical foundations of personality psychology in educational contexts

The Five-Factor Model (FFM) of personality, comprising Neuroticism, Extraversion, Openness to Experience, Agreeableness, and Conscientiousness, represents the dominant framework for conceptualizing and measuring individual differences in personality within educational research (Rodríguez Hernández et al., 2025; Váradi, 2022). Decades of research have established robust relationships between FFM traits and academic outcomes including achievement motivation, academic engagement, and learning strategy use (Rodríguez Hernández et al., 2025). Critically, the mechanisms by which these traits influence learning are understood to operate through both affective and cognitive pathways: traits like Neuroticism predispose individuals to heightened emotional reactivity under evaluative pressure, while Conscientiousness facilitates goal-directed effort regulation (Pekrun et al., 2007). In the context of specialized performance-based disciplines, these trait-outcome relationships may manifest with particular salience, as the cognitive, affective, and motor demands of music learning create conditions in which dispositional tendencies are expressed more visibly (Bogunović, 2024). Recent psychometric innovations, such as those integrating expressive-semantic frameworks (Price, 2026), have further refined our capacity to measure personality constructs with greater contextual sensitivity, reinforcing the importance of domain-specific validation.

Recent research in personality assessment has increasingly focused on context-specific instruments capable of capturing subtle individual differences across educational settings. Rosenblad et al. (2025) contributed to this line of work by proposing the SOLACE Spectrum, a comprehensive personality assessment model for therapeutic and personal growth contexts. Similarly, Guerrero et al. (2025) highlighted the importance of validating personality-related constructs across cultural and linguistic settings through their work on agentic engagement measures. Collectively, these studies suggest that personality assessment should be both theoretically grounded and practically relevant to specific educational contexts.

### 2.2 Learning emotions and achievement motivation theory

The Control-Value Theory (CVT) of achievement emotions (Pekrun et al., 2007) provides the primary theoretical lens for the present study. CVT posits that learning emotions are generated by students’ cognitive appraisals of their perceived control over learning activities and the subjective value they assign to outcomes. Within this framework, personality traits are conceptualized as distal antecedents that systematically shape control and value appraisals, and therefore indirectly influence the emotional experiences that emerge during learning (Pekrun et al., 2007). For instance, students high in Neuroticism may chronically perceive lower control over performance outcomes, predisposing them toward anxiety and shame; conversely, students high in Openness may assign greater value to the intrinsic aesthetic dimensions of music learning, thereby experiencing elevated enjoyment and reduced boredom. This trait-appraisal-emotion pathway constitutes the core theoretical model guiding the present investigation. CVT also distinguishes between positive activating emotions (e.g., enjoyment, hope), positive deactivating emotions (e.g., relief), negative activating emotions (e.g., anxiety, anger), and negative deactivating emotions (e.g., boredom, hopelessness), providing a nuanced taxonomy for examining differential personality-emotion relationships.

Recent studies on learning emotions have emphasized the importance of measurement precision and contextual appropriateness. Research on emotional intelligence and educational performance has provided useful insights into how affective factors mediate academic outcomes. Bogunović (2024) contribute to this literature through a neuropsychological analysis of decision-making and emotional processing, highlighting the complex interplay between cognitive and affective systems. In addition, studies involving specialized populations have underscored the importance of considering individual differences in emotional regulation when examining learning emotions.

### 2.3 Music education psychology and individual differences

Music education occupies a distinctive position within educational psychology because it integrates cognitive, motor, expressive, and social–emotional processes within a single learning domain. As highlighted by Váradi (2022) music learning involves individual practice, public performance, ensemble collaboration, and aesthetic self-expression, all of which can elicit diverse emotional responses and place varying demands on students’ regulatory capacities (Menon et al., 2025).

Performance anxiety is one of the most prominent negative emotions in music education and has important implications for students’ achievement and well-being. Bogunović (2024) emphasizes that personality traits play a significant role in shaping musical outcomes, with neuroticism associated with higher vulnerability to anxiety, while extraversion and conscientiousness are linked to greater motivation, engagement, and performance. Similarly, Huang (2025) demonstrates that the Big Five personality framework provides a useful lens for understanding learning processes and emotional regulation in music education contexts.

Despite these contributions, research examining the relationship between personality traits and a broader range of learning emotions in music education remains limited. Most existing studies focus on specific aspects such as socio-emotional development or motivation, rather than systematically investigating how personality shapes emotional experiences across different music learning contexts, particularly in non-Western settings.

These distinctive features of music education provide a valuable context for examining personality–emotion relationships. Unlike conventional academic subjects, music learning combines cognitive processing with expressive performance, allowing personality traits to manifest more visibly in emotional experiences. Although advances in psychometric methods have improved the assessment of personality in educational settings, systematic research on personality–emotion relations within music education remains limited.

### 2.4 Psychometric assessment and validation in educational research

Psychometric evaluation is a foundation of effective educational research, providing a methodological basis for reliable and valid measurement in the assessment of psychological constructs. Modern psychometric theory emphasizes comprehensive validation procedures that involve multiple types of evidence, including content validity, construct validity, and criterion-related validity. Vispoel et al. (2024) further contributed to the field by applying multivariate structural equation modeling to examine reliability and measurement error in educational assessment contexts. These methodological advances have important implications for studies investigating complex relationships between personality and educational outcomes.

Psychometric validation should be conducted with careful consideration of both statistical rigor and practical applicability. Recent studies have highlighted the importance of cultural adaptation and cross-linguistic validation, demonstrating the role of context in the use of personality assessment tools across diverse populations and settings. Psychometric principles have also been applied in related areas, such as the measurement of moral inclinations by Chen Y. et al. (2024), the validation of character strengths by Varela-Figueroa et al. (2025), and the study of behavioral addiction patterns, illustrating the versatility of psychometric approaches in psychological research. These developments reinforce the importance of psychometric rigor in advancing our understanding of relationships between personality and emotions in educational contexts.

Table 1 presents the methodological features, sample characteristics, measurement instruments, and main findings of ten research papers published between 2024 and 2026, providing a systematic comparison of recent research approaches. This comparative analysis highlights the diversity of methods currently used to study personality and emotion across different contexts.

**Table 1:** Comparative table of the study.

Author(s) and year	Sample size	Context	Assessment tools	Key findings
Price (2026)	185	General	Drawmetrics, IPIP-NEO 120	Successful embedding-based mapping between expressive-semantic framework and Big Five model
Rosenblad et al. (2025)	342	Therapy	SOLACE Spectrum	Validated personality assessment tool for therapeutic growth contexts
Guerrero et al. (2025)	456	University	AES, EVAES	Cross-cultural validation of agentic engagement scales in Spanish context
Rodríguez Hernández et al. (2025)	298	Education	Emotional Competence Model	Extended framework beyond emotional intelligence for teacher competence
Glaveanu and Maier (2025)	234	University	Big Five, AI Attitude Scale	Personality traits influence attitudes toward AI in education
Bogunović (2024)	120	Laboratory	fNIRS, Personality Scales	Neurological correlates of personality in decision-making under risk
Zmork-Martínez et al. (2025)	387	Clinical	SI-Bord Chinese version	Cross-cultural validation of borderline personality screening instrument
Varela-Figueroa et al. (2025)	523	University	VIA-72 Spanish Version	Psychometric validation of character strengths inventory in Spanish context
Huang (2025)	635	Gaming	PPDS Scale	Validation of player personality assessment in educational gamification contexts
Vispoel et al. (2024)	892	Psychometric	SEM techniques	Advanced statistical methods for reliability assessment in hierarchical constructs

### 2.5 Research gap and justification for current study

A systematic review of the literature reveals three critical gaps that justify the present investigation. First, while Big Five personality traits have been extensively studied in relation to academic achievement and learning strategy use across general educational settings, their predictive relationships with domain-specific learning emotions in music education have not been empirically examined. The few existing studies on personality in music education have focused primarily on performance anxiety and achievement motivation, with limited attention given to the full spectrum of achievement emotions captured by the AEQ framework (Pekrun et al., 2007). Second, although the AEQ-S (Bieleke et al., 2021) has demonstrated strong psychometric properties in general educational samples, its validity in the specialized context of music learning has not been established. The unique demands of music education may affect the factor structure, item loadings, and criterion-related validity of the instrument relative to its original validation context. Third, the Chinese music education context has received limited attention in the international personality–emotion literature, raising questions about the cross-cultural applicability of findings derived from Western samples (Gross, 2015).

Moreover, although validated personality assessment tools and learning emotion measures are available, there are no studies that have examined their relevance and psychometric properties in music education settings. Table 1 developed above shows that research conducted to date has mostly concentrated on general educational and therapeutic environments or those involving specialist populations, but none at the level of music learning. This is a major shortcoming in our understanding of how basic psychological processes may differ in creative and performance-based learning settings.

Furthermore, the methodological strategies adopted in past studies have predominantly relied on conventional statistical methods, lacking a complex, multivariate perspective on personality–emotion correlations in education. Although more advanced psychometric analyses have become available in recent years, including those by Vispoel et al., their application in music education studies has not been examined. The present study addresses these gaps by presenting a systematic exploration of personality–emotion correlations in music education settings, using validated measures and advanced analytical methods to generate evidence of both theoretical and practical significance (Huprich et al., 2024).

Overall, prior studies confirm general relationships between personality traits and academic outcomes; however, limited research has examined these relationships in music education using validated psychometric models. This gap justifies the present study.

## 3 Methodology

This section describes the research methodology employed to examine the relationships between Big Five personality traits and learning emotions among Chinese undergraduate music students. A cross-sectional quantitative design was adopted, integrating psychometric validation procedures with structural equation modeling to simultaneously address measurement quality and substantive research questions. The study recruited 298 undergraduate music students from three geographically distributed Chinese universities. Participants were assessed using two validated instruments: the Chinese Big Five Personality Inventory-Short Form (CBF-PI-SF) and an adapted version of the Achievement Emotions Questionnaire-Short (AEQ-S) contextualized for the music learning environment. Data were analyzed using IBM SPSS Statistics 27.0 and AMOS 27.0. Construct validity was evaluated via confirmatory factor analysis (CFA), and predictive relationships were examined using structural equation modeling (SEM). Ethical approval was obtained from the relevant Institutional Review Board prior to data collection, as detailed in Section 3.6.

### 3.1 Research design

This study employed a cross-sectional quantitative design to examine the relationships between Big Five personality traits and learning emotions among Chinese undergraduate music students. The cross-sectional method was chosen because it enables the collection of data from a large number of participants at a single point in time with minimal effort and allows for the analysis of associations between personality constructs and emotions in the context of music learning (Creswell and Creswell, 2018). The research design combines psychometric validation processes with correlational analysis to address both measurement quality and substantive research questions.

A reflective measurement model was used, where observed variables represent underlying latent constructs (Jarvis et al., 2003). This approach is theoretically appropriate for personality traits and learning emotions, which are generally considered to give rise to their observable behavioral and self-report manifestations rather than being shaped by them. The study design employs multiple analytical approaches, including confirmatory factor analysis (CFA) to test construct validity and structural equation modeling (SEM) to examine predictive relationships.

### 3.2 Participants and sampling

The target population comprised undergraduate students majoring in music at Chinese universities. Participants were recruited from three comprehensive universities located in different regions of China to enhance sample representativeness. The inclusion criteria were: (a) respondents should be full-time undergraduate students, (b) students should be majoring in music education, music performance, or musicology, and (c) students should be at least 18 years old and willing to provide informed consent to participate.

A total of 347 questionnaires were distributed, and 312 were returned (response rate: 89.9%). Participants who responded carelessly or submitted incomplete responses were excluded based on attention check items, resulting in a final analytical sample of 298 valid responses. The sample is adequate to meet the minimum requirements of structural equation modeling, which suggest that a sample of 200 or 10–20 respondents per parameter should be used (Chen Q. et al., 2024).

Table 2 shows the demographic characteristics of the participants (*N* = 298). The sample was predominantly female (60.4%), with most participants aged between 18 and 23 years (92.7%). There was a relatively even distribution across academic years, with sophomores representing the largest group (29.9%). Most participants majored in music education (52.3%), followed by music performance (32.9%) and musicology (14.8%). The majority of participants (56.4%) had 5–10 years of music training experience, indicating substantial prior musical experience.

**Table 2:** Demographic characteristics of participants (*N* = 298).

Characteristic	Category	*n*	%
Gender	Male	118	39.6
	Female	180	60.4
Age (years)	18–20	142	47.7
	21–23	134	45.0
	24+	22	7.3
Year of study	Freshman	68	22.8
	Sophomore	89	29.9
	Junior	82	27.5
	Senior	59	19.8
Major specialization	Music education	156	52.3
	Music performance	98	32.9
	Musicology	44	14.8
Years of music training	<5 years	52	17.4
	5–10 years	168	56.4
	>10 years	78	26.2

### 3.3 Measurement instruments

#### 3.3.1 Big Five personality traits

The Chinese Big Five Personality Inventory-Short Form (CBF-PI-SF) was used as a measure of personality traits and was designed and validated by Marcos et al. The CBF-PI-SF consists of 40 items assessing five personality scales, namely: Neuroticism (8 items), Extraversion (8 items), Openness to Experience (8 items), Agreeableness (8 items), and Conscientiousness (8 items). Each item is rated on a 6-point Likert scale ranging from 1 (strongly disagree) to 6 (strongly agree). The CBF-PI-SF has demonstrated good psychometric properties in Chinese populations, with Cronbach’s alpha ranging from 0.76 to 0.81 across the subscales (Marcos et al., 2023).

#### 3.3.2 Learning emotions in music education

Learning emotions were assessed using an adapted version of the Achievement Emotions Questionnaire-Short (AEQ-S) (Bieleke et al., 2021). The adaptation process followed a rigorous, multi-step methodological protocol to ensure content validity and construct equivalence within the music education context. Specifically, the adaptation involved three sequential phases: (1) forward translation and semantic adjustment of all items to replace generic academic references with music-specific learning scenarios (e.g., “during class” was replaced with “during music practice or rehearsal”); (2) expert panel review by three music education specialists and two educational psychologists who independently rated each item for content relevance and contextual appropriateness on a 4-point scale (Content Validity Index = 0.89); and (3) cognitive interviews with a pilot sample of 15 music students to verify item comprehensibility and ecological validity. Items that received expert ratings below the acceptable threshold or were flagged as ambiguous by more than two participants were revised accordingly. For example, the Enjoyment subscale item ‘I enjoy being in class’ was adapted to ‘I enjoy being in music practice or rehearsal’; the Anxiety item ‘I get nervous during exams’ was adapted to ‘I get nervous before or during music performances or assessments’. The final adapted scale retained the original eight-factor structure, comprising eight discrete learning emotion subscales assessed across three temporal phases of music learning activities (before, during, and after): Enjoyment (4 items), Hope (4 items), Pride (4 items), Anger (4 items), Anxiety (4 items), Shame (4 items), Hopelessness (4 items), and Boredom (4 items). All items were rated on a 5-point Likert scale ranging from 1 (not at all) to 5 (very much). The adapted instrument demonstrated strong psychometric properties in the present sample, as reported in the Section 4 (Table 3).

**Table 3:** Summary of measurement instruments.

Instrument	Constructs	Items	Scale	Source
CBF-PI-SF	Big Five traits	40	6-point	Marcos et al. (2023)
AEQ-S (adapted)	Learning emotions	32	5-point	Bieleke et al. (2021)
Demographics	Background info	8	Various	Researcher-developed

### 3.4 Data collection procedures

Data were collected between September and November 2025 using both online and paper-based questionnaire administration modalities. Online data collection was conducted via the Wenjuanxing platform,^1^ a widely used Chinese survey tool with built-in data security protocols, including IP-based duplicate response detection and response-time monitoring. Paper-based questionnaires were administered in standardized classroom settings by trained research assistants who followed a scripted protocol to ensure consistent administration conditions across all participating universities. Both modalities presented identical item content, response formats, and instructions. To ensure methodological comparability, equivalence testing between online and paper-based responses was performed prior to pooling, confirming no significant differences in mean scores or factor structures across administration modes (all *p* > 0.20). Specifically, independent-samples t-tests revealed no significant mean differences between online (*n* = 187) and paper-based (*n* = 111) respondents on any personality or emotion subscale (all *p* > 0.20). Multigroup CFA further confirmed configural and metric invariance across administration modes (ΔCFI < 0.01), supporting the pooling of responses. The questionnaire packet included (a) an informed consent form; (b) the CBF-PI-SF (40 items); (c) the adapted AEQ-S (32 items); and (d) a demographic background questionnaire (8 items), presented in this fixed order. Estimated completion time was 15–20 min.

Participants were informed of the study objectives and told that participation was voluntary, that they could withdraw at any time, and that their responses would remain confidential. Informed consent was obtained prior to questionnaire completion. Completion time averaged 15–20 min. Data quality was ensured through the inclusion of three attention check items at various points in the questionnaire, and responses that failed more than one attention check were excluded from analysis.

### 3.5 Data analysis strategy

All analyses were conducted using IBM SPSS Statistics 27.0 and IBM SPSS AMOS 27.0. The analytical sequence comprised four phases. Phase 1 involved preliminary data screening: missing data patterns were evaluated using Little’s MCAR test (all *p* > 0.10), and missing values (< 2% per variable) were imputed using the Expectation–Maximization (EM) algorithm as implemented in SPSS. Univariate outliers were identified as cases with |z| > 3.29 on any variable; multivariate outliers were identified via Mahalanobis distance (*p* < 0.001). Normality was assessed via skewness and kurtosis values, with acceptable thresholds of |skew| < 2.0 and |kurt| < 7.0 (Fornell and Larcker, 1981). Phase 2 assessed internal consistency reliability using Cronbach’s alpha (*α* ≥ 0.70 criterion) (Pekrun et al., 2007) and composite reliability (CR ≥ 0.70 criterion) (Hu and Bentler, 1999). Phase 3 employed confirmatory factor analysis (CFA) using maximum likelihood estimation to evaluate the factorial validity of both measurement models.

Model fit was assessed using several indices, including the chi-square statistic (χ^2^), Comparative Fit Index (CFI > 0.90), Tucker–Lewis Index (TLI > 0.90), Root Mean Square Error of Approximation (RMSEA < 0.08), and Standardized Root Mean Square Residual (SRMR < 0.08) (Hu and Bentler, 1999). Phase 4 involved structural equation modeling (SEM) to examine the predictive relationships between personality traits and learning emotions. Convergent validity was assessed using Average Variance Extracted (AVE > 0.50) and Composite Reliability (CR > 0.70), and discriminant validity was evaluated by comparing the square root of AVE with inter-construct correlations (Fornell and Larcker, 1981).

### 3.6 Ethical considerations

This study was approved by the Institutional Review Board of the university where the lead researcher is enrolled (Approval No.: XHLL-2026-K010). All procedures complied with the Declaration of Helsinki and the American Psychological Association Code of Ethics for research involving human participants. Participation was voluntary, and informed consent was obtained from all participants. No personally identifiable data were collected, and all data were stored on password-secured servers accessible only to the research team.

## 4 Results

The section highlights the results of psychometric validation and structural equation modeling examining personality–emotion relationships among Chinese music students (*N* = 298). Data analyses were conducted sequentially: preliminary data screening indicated good data quality with minimal missing values and acceptable normality; reliability analyses showed that all subscales had strong internal consistency (*α* = 0.77 to 0.87); confirmatory factor analysis established good factorial validity of both instruments with acceptable fit indices; and convergent and discriminant validity were supported by AVE values and the Fornell–Larcker criterion. The structural equation model showed that personality traits collectively accounted for substantial variance in positive emotions (R^2^ = 0.42), negative emotions (R^2^ = 0.38), and boredom (R^2^ = 0.29), with extraversion, openness, neuroticism, and conscientiousness emerging as significant predictors.

### 4.1 Preliminary analyses

Data screening prior to the main analyses included missing values, outliers, and normality. The proportion of missing data was very low (less than 2% for all variables) and was handled using the Expectation–Maximization (EM) algorithm. Standardized scores (|z| > 3.29) were used to detect univariate outliers, and Mahalanobis distance was used to detect multivariate outliers. Five cases were identified as multivariate outliers and were retained after inspection, as no data entry errors were found. Normality was assessed using skewness and kurtosis values, with all variables falling within acceptable ranges (|skewness| < 2, |kurtosis| < 7) (Curran et al., 1996).

Table 4 presents descriptive statistics and reliability coefficients for all study variables. Among personality traits, openness (*M* = 4.28) and extraversion (*M* = 4.02) showed the highest means, while neuroticism was the lowest (*M* = 3.21). For learning emotions, positive emotions (enjoyment, hope, pride) scored above the scale midpoint, whereas negative emotions remained below the midpoint, with hopelessness (*M* = 2.08) and anger (*M* = 2.15) being the lowest. All variables demonstrated acceptable normality (|skewness| < 2, |kurtosis| < 7) and strong internal consistency reliability (*α* = 0.77 to 0.87).

**Table 4:** Descriptive statistics for all study variables (*N* = 298).

Variable	M	SD	Skew	Kurt	α
Personality traits					
Neuroticism	3.21	0.89	0.18	−0.42	0.84
Extraversion	4.02	0.76	−0.31	0.15	0.81
Openness	4.28	0.72	−0.45	0.28	0.79
Agreeableness	4.15	0.68	−0.22	−0.18	0.77
Conscientiousness	3.89	0.81	−0.15	−0.31	0.82
Positive learning emotions					
Enjoyment	3.78	0.82	−0.52	0.21	0.86
Hope	3.65	0.79	−0.38	0.12	0.83
Pride	3.42	0.91	−0.28	−0.35	0.85
Negative learning emotions					
Anxiety	2.89	0.94	0.35	−0.28	0.87
Anger	2.15	0.88	0.72	0.45	0.82
Shame	2.34	0.86	0.58	0.22	0.81
Hopelessness	2.08	0.92	0.85	0.52	0.84
Boredom	2.28	0.90	0.62	0.18	0.80

M, Mean; SD, Standard Deviation; Skew, Skewness; Kurt, Kurtosis; α, Cronbach’s alpha.

**Figure 1:**
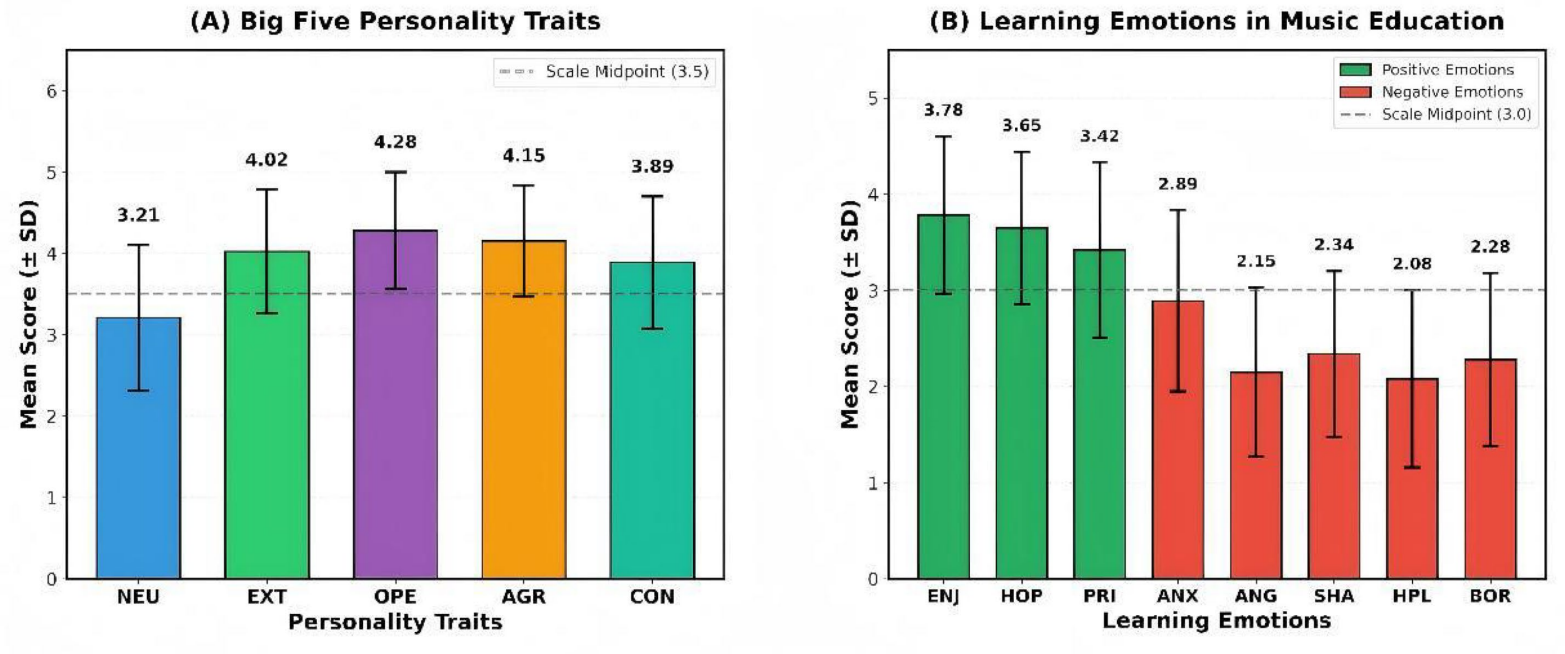
Descriptive statistics: mean scores with standard deviations for Big Five personality traits and learning emotions among Chinese music students (*N* = 298). Panel **(A)** displays mean scores (±SD) for the five personality traits (CBF-PI-SF, 6-point scale; dashed line = midpoint 3.5). Openness (*M* = 4.28) and Extraversion (*M* = 4.02) scored above midpoint, while Neuroticism (*M* = 3.21) was lowest, indicating a generally adaptive personality profile. Panel **(B)** shows mean scores (±SD) for eight learning emotions (AEQ-S adapted, 5-point scale; dashed line = midpoint 3.0). Positive emotions (Enjoyment *M* = 3.78, Hope *M* = 3.65, Pride *M* = 3.42) all exceeded the midpoint, whereas negative emotions (Anxiety, Anger, Shame, Hopelessness, Boredom) fell below midpoint, reflecting a predominantly positive emotional climate in this music student sample. Green bars = positive emotions; red bars = negative emotions. Error bars represent ±1 SD.

The descriptive statistics for personality traits and learning emotions are visually presented in Figure 1, which presents the mean scores and standard deviations for Big Five personality traits (Panel A) and learning emotions (Panel B) among Chinese music students (*N* = 298). In Panel A, music students scored highest on Openness (*M* = 4.28) and Extraversion (*M* = 4.02), both above the scale midpoint (3.5), reflecting the creative and socially expressive nature typical of music learners. Neuroticism had the lowest mean (*M* = 3.21), indicating that the sample was relatively stable in terms of emotional tendencies. In Panel B, positive emotions (Enjoyment: *M* = 3.78, Hope: *M* = 3.65, Pride: *M* = 3.42) were rated above the midpoint of the scale (3.0), indicating that the emotional state of music learners was generally positive. In contrast, negative emotions remained below the midpoint, with the lowest scores observed for Hopelessness (*M* = 2.08) and Anger (*M* = 2.15), suggesting that music learners did not experience high levels of negative emotions in their learning.

### 4.2 Reliability analysis

Cronbach’s alpha coefficients were used to assess internal consistency reliability. Table 4 shows that all subscales exhibited acceptable to excellent reliability, with alpha values ranging from 0.77 (Agreeableness) to 0.87 (Anxiety). For personality traits, Cronbach’s alpha coefficients ranged from 0.77 to 0.84, consistent with prior validation studies of the CBF-PI-SF (Marcos et al., 2023). For learning emotions, alpha coefficients ranged from 0.80 to 0.87, indicating that the adapted AEQ-S demonstrated good internal consistency in the music learning context.

In addition, composite reliability (CR) was calculated as a complementary measure of reliability. CR values ranged from 0.78 to 0.89, exceeding the recommended threshold of 0.70 (Hair, 2009), further supporting the reliability of the measurement instruments used in the current sample.

### 4.3 Confirmatory factor analysis

#### 4.3.1 Measurement model for personality traits

A confirmatory factor analysis was conducted to evaluate the factorial validity of the five-factor structure of the CBF-PI-SF. The initial model demonstrated acceptable fit to the data: χ2 (730) = 1285.42, *p* < 0.001, CFI = 0.912, TLI = 0.904, RMSEA = 0.051 (90% CI: 0.046, 0.055), SRMR = 0.058. All standardized factor loadings were statistically significant (p<.001) and ranged from 0.52 to 0.78, exceeding the minimum threshold of 0.40. These results support the five-factor structure of personality traits in the present sample of music students.

#### 4.3.2 Measurement model for learning emotions

The eight-factor structure of the adapted AEQ-S was evaluated through CFA. The model exhibited good fit: χ^2^(436) = 892.15, *p* < 0.001, CFI = 0.928, TLI = 0.918, RMSEA = 0.059 (90% CI: 0.054, 0.065), SRMR = 0.052. Standardized factor loadings ranged from 0.58 to 0.84, all statistically significant (*p* < 0.001). The results confirm that the adapted learning emotions measure maintains adequate factorial validity in the music education context.

Table 5 presents the confirmatory factor analysis fit indices for the measurement models. The five-factor personality model demonstrated acceptable fit (CFI = 0.912, TLI = 0.904, RMSEA = 0.051, SRMR = 0.058), as did the eight-factor learning emotions model (CFI = 0.928, TLI = 0.918, RMSEA = 0.059, SRMR = 0.052). The full measurement model also achieved satisfactory fit (CFI = 0.905, RMSEA = 0.045, SRMR = 0.062). Most models met or closely approached established thresholds (CFI/TLI > 0.90; RMSEA/SRMR < 0.08). Although the TLI values for the full measurement model and structural model were marginally below the conventional 0.90 threshold, the remaining fit indices were within acceptable ranges, supporting the adequacy of the overall model fit.

**Table 5:** Confirmatory factor analysis fit indices.

Model	χ^2^ (df)	CFI	TLI	RMSEA [90% CI]	SRMR
Big Five (5-factor)	1285.42 (730)	0.912	0.904	0.051 [0.046, 0.055]	0.058
Learning emotions (8-factor)	892.15 (436)	0.928	0.918	0.059 [0.054, 0.065]	0.052
Full measurement model	2456.78 (1548)	0.905	0.898	0.045 [0.042, 0.048]	0.062
Acceptable thresholds	p < 0.05	> 0.90	> 0.90	< 0.08	< 0.08

CFI, Comparative Fit Index; TLI, Tucker-Lewis Index; RMSEA, Root Mean Square Error of Approximation; CI, Confidence Interval; SRMR, Standardized Root Mean Square Residual.

**Figure 2:**
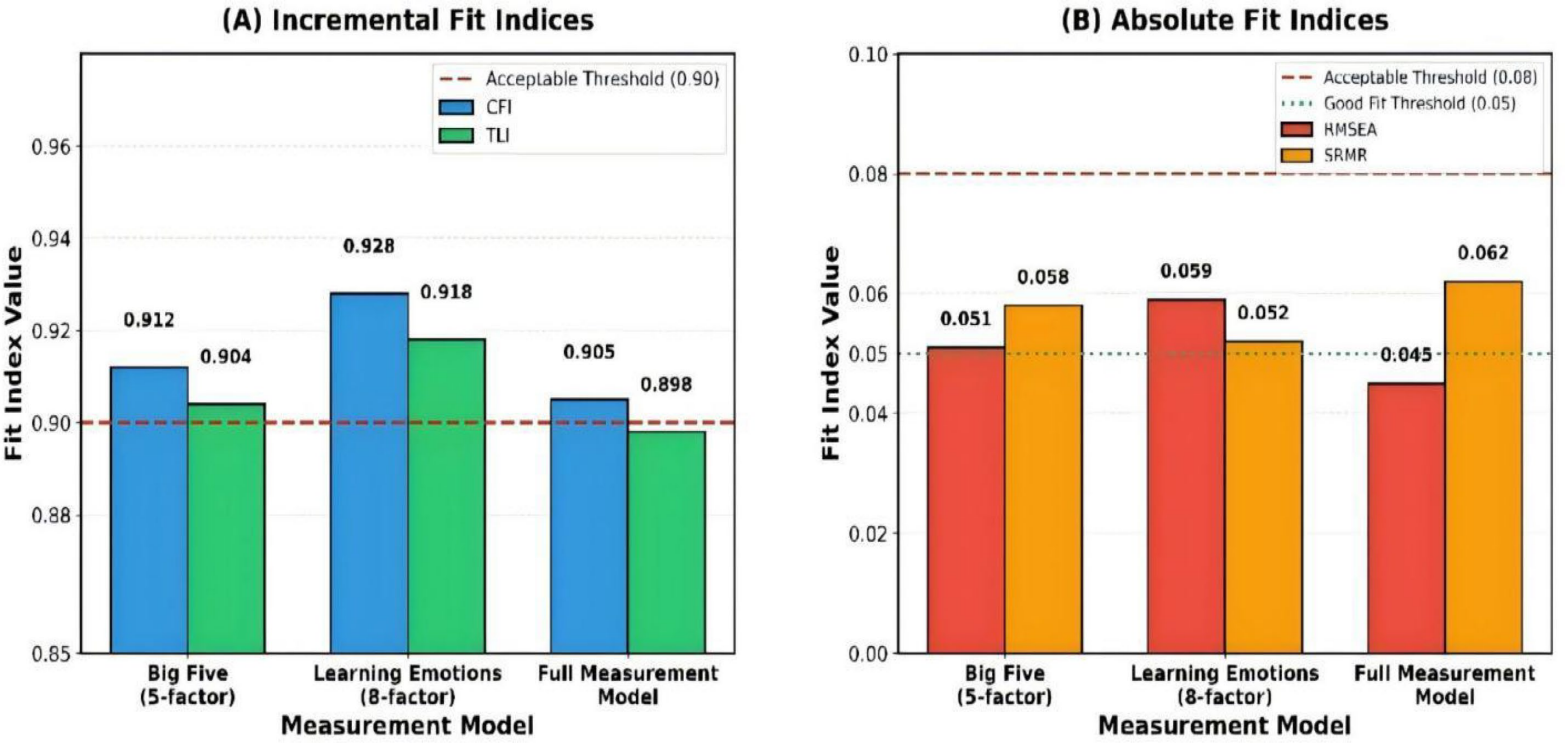
Confirmatory factor analysis: model fit indices comparison across three measurement models. Panel **(A)** presents incremental fit indices (CFI and TLI) for the Big Five 5-factor model (CFI = 0.912, TLI = 0.904), the Learning Emotions 8-factor model (CFI = 0.928, TLI = 0.918), and the Full Measurement Model (CFI = 0.905, TLI = 0.898). The red dashed line marks the acceptable threshold of 0.90; all models meet or approach this threshold. Panel **(B)** displays absolute fit indices (RMSEA and SRMR) for the same three models. The red dashed line marks the acceptable threshold of 0.08; the teal dotted line marks the good-fit threshold of 0.05. All RMSEA and SRMR values fall well below 0.08, confirming acceptable absolute fit across all models. The Full Measurement Model achieved the lowest RMSEA (0.045), approaching excellent fit.

The confirmatory factor analysis (CFA) fit indices in the three measurement models are depicted in Figure 2. Panel A shows the incremental fit indices (CFI and TLI), with two models above the conventional 0.90 threshold and the full measurement model approaching that threshold (TLI = 0.898), with the learning emotions model showing the highest level of fit (CFI = 0.928, TLI = 0.918). In Panel B, absolute fit indices (RMSEA and SRMR) are reported; all values are below the acceptable threshold of 0.08, indicating a good fit between the models and the data. It is worth noting that the full measurement model obtained the lowest RMSEA (0.045), which is close to an excellent fit. These findings indicate that the Big Five personality traits and learning emotions measurement instruments demonstrate adequate factorial validity when applied to Chinese music students.

### 4.4 Convergent and discriminant validity

Convergent validity was assessed using Average Variance Extracted (AVE). Table 6 indicates that AVE values for all constructs ranged from 0.51 to 0.62, exceeding the minimum threshold of 0.50 (Fornell and Larcker, 1981). Together with acceptable factor loadings and composite reliability values, these findings provide evidence of sufficient convergent validity.

**Table 6:** Correlation matrix with AVE and CR values.

Variable	1	2	3	4	5	6	7	8	CR	AVE
1. NEU	0.73								0.85	0.53
2. EXT	−0.38**	0.72							0.83	0.52
3. OPE	−0.22**	0.45**	0.71						0.81	0.51
4. AGR	−0.28**	0.32**	0.35**	0.72					0.78	0.52
5. CON	−0.31**	0.29**	0.42**	0.38**	0.74				0.84	0.55
6. POS-E	−0.35**	0.52**	0.48**	0.28**	0.41**	0.79			0.89	0.62
7. NEG-E	0.54**	−0.42**	−0.25**	−0.18**	−0.32**	−0.48**	0.76		0.87	0.58
8. BOR	0.32**	−0.38**	−0.45**	−0.15*	−0.29**	−0.52**	0.58**	0.75	0.82	0.56

NEU, Neuroticism; EXT, Extraversion; OPE, Openness; AGR, Agreeableness; CON, Conscientiousness; POS-E, Positive Emotions; NEG-E, Negative Emotions; BOR, Boredom; CR, Composite Reliability; AVE, Average Variance Extracted. Diagonal, square root of AVE. *p < 0.05; **p < 0.01.

Discriminant validity was assessed using the Fornell–Larcker criterion, whereby the square root of AVE for each construct should be greater than its correlations with other constructs. As shown in Table 5, all diagonal values (square roots of AVE) were higher than the off-diagonal values (inter-construct correlations), supporting the discriminant validity of the study constructs.

Table 6 presents the correlation coefficients alongside composite reliability (CR) and average variance extracted (AVE) values. Each construct demonstrated sufficient convergent validity, with AVE ranging from 0.51 to 0.62 (above 0.50) and CR ranging from 0.78 to 0.89 (above 0.70). Discriminant validity was also established, as the square roots of AVE (0.71 to 0.79) exceeded the corresponding inter-construct correlations. Negative emotions (*r* = 0.54) and boredom (*r* = 0.32) were positively associated with neuroticism, whereas positive emotions were positively related to extraversion (*r* = 0.52) and openness (*r* = 0.48).

**Figure 3:**
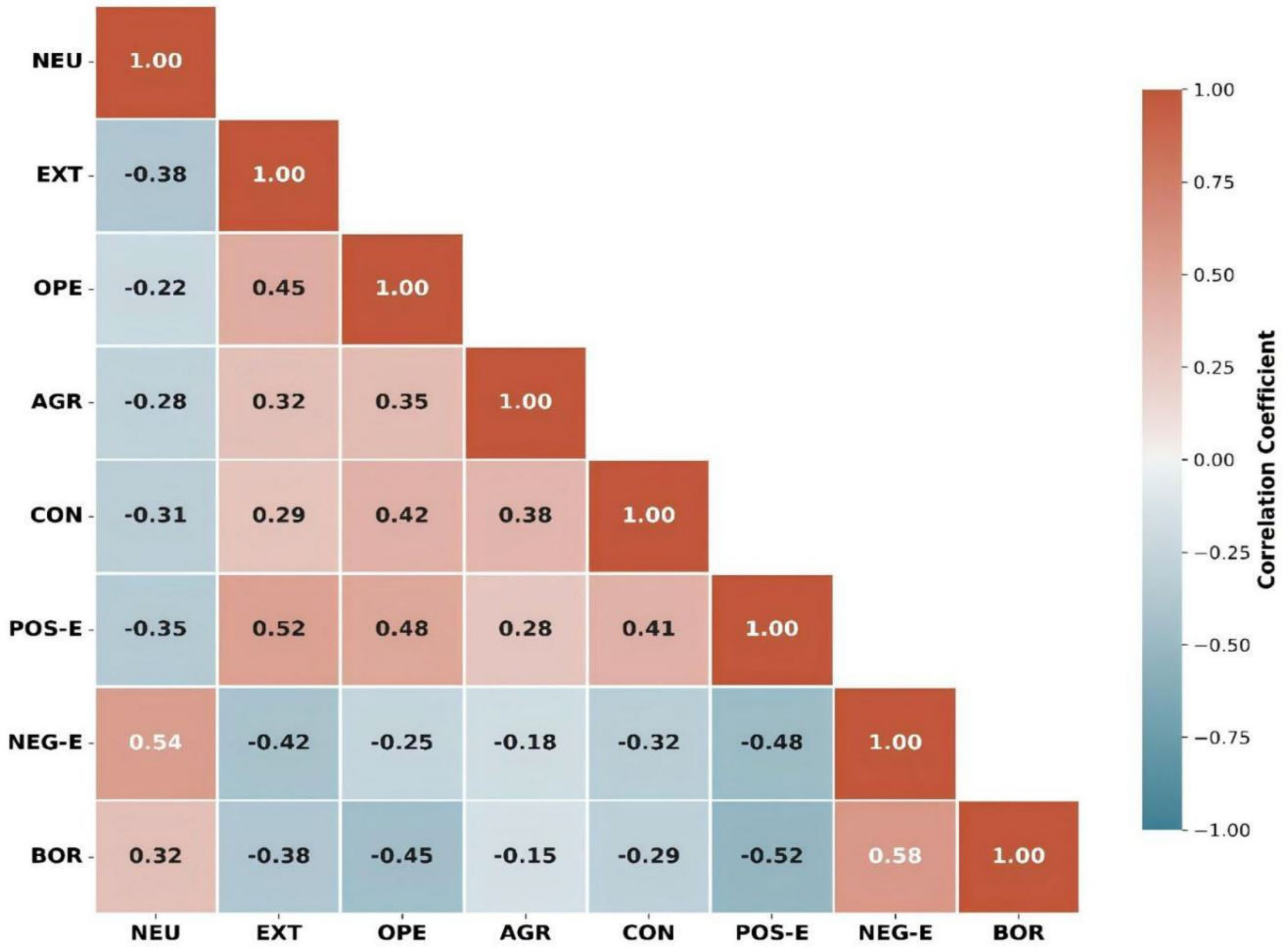
Correlation matrix heatmap: bivariate relationships among Big Five personality traits and learning emotion composites (*N* = 298). The lower-triangular heatmap displays Pearson correlation coefficients for all eight study variables. Cell color encodes both direction and magnitude: warm red tones indicate positive correlations and cool blue tones indicate negative correlations, with color saturation proportional to effect size as shown in the right-hand scale bar (range: −1.00 to +1.00). Key findings: Neuroticism showed the strongest positive association with negative emotions (*r* = 0.54, *p* < 0.01) and boredom (*r* = 0.32, *p* < 0.01); Openness showed the strongest inverse association with boredom (*r* = −0.45, *p* < 0.01); and Extraversion showed the strongest positive association with positive emotions (*r* = 0.52, *p* < 0.01). Negative emotions and boredom were strongly positively correlated (*r* = 0.58, *p* < 0.01), while positive emotions showed inverse relationships with both negative emotions (*r* = −0.48) and boredom (*r* = −0.52). All off-diagonal values marked *p < 0.05 or **p < 0.01.

Figure 3 shows the correlations between the Big Five personality traits and learning emotions in the form of a heat map with color-coded values. Blue indicates positive correlations, and red indicates negative correlations, with color intensity reflecting the strength of the correlations. Neuroticism was most strongly positively correlated with negative emotions (*r* = 0.54) and boredom (*r* = 0.32), whereas extraversion and openness were strongly correlated with positive emotions (*r* = 0.52 and *r* = 0.48, respectively). Notably, negative emotions and boredom were strongly positively correlated (*r* = 0.58), while positive emotions showed inverse relationships with both negative emotions (*r* = −0.48) and boredom (*r* = −0.52). These patterns confirm theoretically expected relationships, with adaptive personality traits (extraversion, openness, conscientiousness) linked to positive emotional experiences, and neuroticism associated with maladaptive emotional outcomes in music learning contexts.

### 4.5 Structural equation modeling results

Structural equation modeling was conducted to examine the predictive relationships between Big Five personality traits and learning emotions. The structural model demonstrated acceptable fit: χ^2^(1625) = 2678.45, *p* < 0.001, CFI = 0.902, TLI = 0.895, RMSEA = 0.047 (90% CI: 0.044, 0.050), SRMR = 0.065. The results of the path analysis are presented in Table 7.

**Table 7:** Structural model path coefficients.

Path	β	SE	t	*p*
Paths to positive emotions				
Neuroticism → positive emotions	−0.18	0.05	−3.62	<0.001
Extraversion → positive emotions	0.35	0.06	5.89	<0.001
Openness → positive emotions	0.28	0.05	5.12	<0.001
Agreeableness → positive emotions	0.08	0.05	1.52	0.129
Conscientiousness → positive emotions	0.21	0.05	4.18	<0.001
Paths to negative emotions				
Neuroticism → negative emotions	0.42	0.05	8.15	<0.001
Extraversion → negative emotions	−0.22	0.06	−3.85	<0.001
Openness → negative emotions	−0.05	0.05	−0.98	0.328
Agreeableness → negative emotions	0.04	0.05	0.78	0.436
Conscientiousness → negative emotions	−0.15	0.05	−2.95	0.003
Paths to boredom				
Neuroticism → boredom	0.18	0.05	3.45	<0.001
Extraversion → boredom	−0.15	0.06	−2.62	0.009
Openness → boredom	−0.32	0.05	−6.25	<0.001
Agreeableness → boredom	0.02	0.05	0.38	0.702
Conscientiousness → boredom	−0.12	0.05	−2.28	0.023

β, Standardized path coefficient; SE, Standard Error. Model fit: χ^2^(1625) = 2678.45, CFI = 0.902, TLI = 0.895, RMSEA = 0.047, SRMR = 0.065.

**Figure 4:**
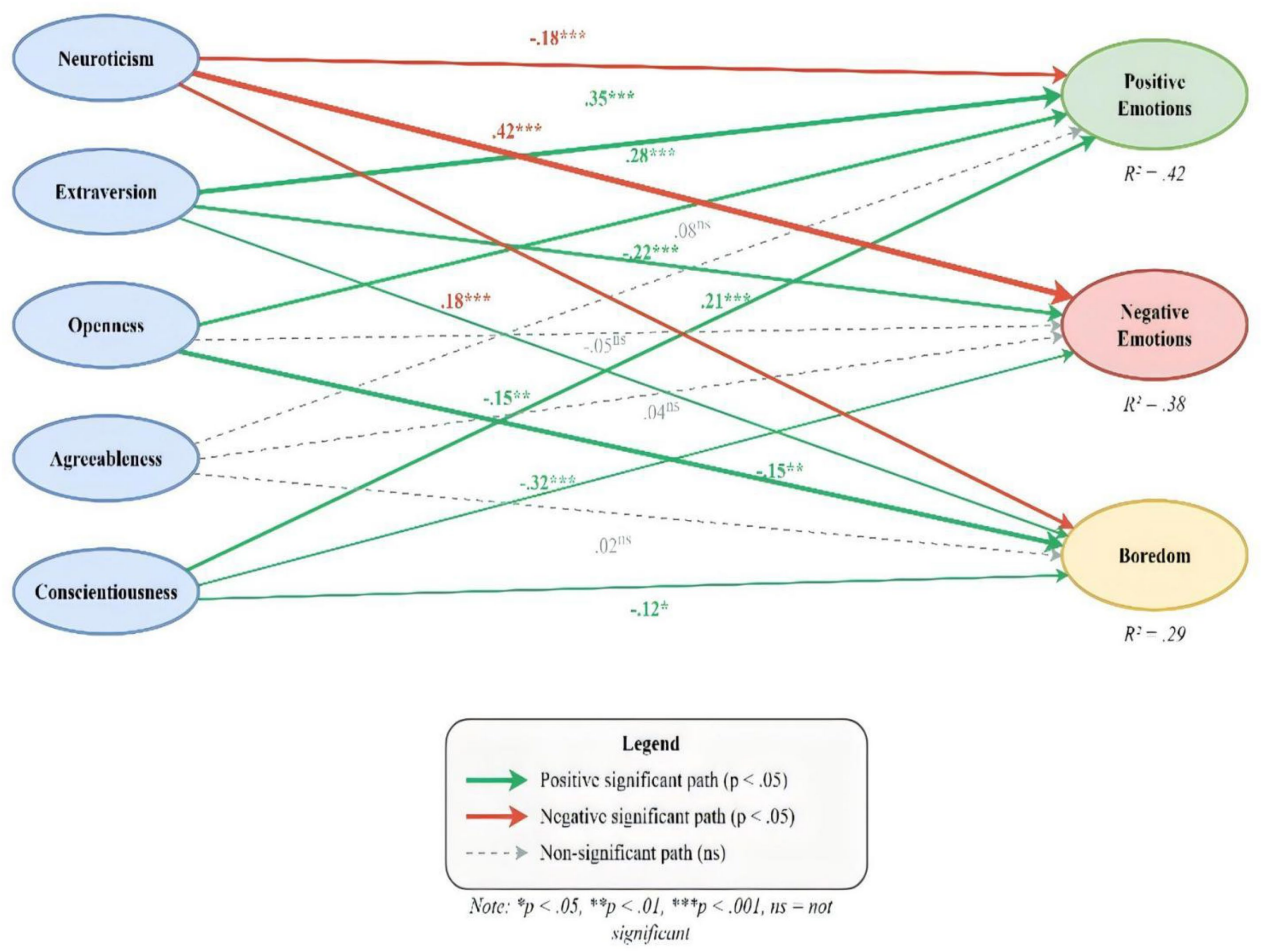
Structural equation model: Standardized path coefficients (β) from Big Five personality traits to learning emotion outcomes (*N* = 298). Predictor variables (Big Five personality traits) are displayed in blue ellipses on the left; outcome variables are displayed in colored ellipses on the right (green = positive emotions, pink = negative emotions, yellow = boredom). Solid green arrows indicate statistically significant positive paths (*p* < 0.05); solid red arrows indicate statistically significant negative paths (*p* < 0.05); dashed gray arrows indicate non-significant paths (*p* > 0.05). Standardized path coefficients (β) are displayed adjacent to each arrow. R^2^ values indicate variance explained in each outcome: positive emotions R^2^ = 0.42, negative emotions R^2^ = 0.38, boredom R^2^ = 0.29. Model fit: χ^2^(1625) = 2678.45, CFI = 0.902, TLI = 0.895, RMSEA = 0.047, SRMR = 0.065. *p < 0.05, **p < 0.01, ***p < 0.001, ns = not significant.

Figure 4 illustrates the structural equation model depicting standardized path coefficients (β) from Big Five personality traits to three learning emotion outcomes among Chinese music students. The model demonstrates that personality traits collectively explained substantial variance in positive emotions (R^2^ = 0.42), negative emotions (R^2^ = 0.38), and boredom (R^2^ = 0.29).

Among the significant paths, extraversion (*β* = 0.35, *p* < 0.001) and openness (*β* = 0.28, *p* < 0.001) emerged as the strongest positive predictors of positive learning emotions, while conscientiousness also showed a significant positive effect (*β* = 0.21, *p* < 0.001). Neuroticism was the dominant predictor of negative emotions (*β* = 0.42, *p* < 0.001) and positively predicted boredom (*β* = 0.18, *p* < 0.001), whereas it negatively predicted positive emotions (*β* = −0.18, *p* < 0.001). Notably, openness demonstrated the strongest protective effect against boredom (*β* = −0.32, *p* < 0.001), followed by extraversion (*β* = −0.15, *p* < 0.01) and conscientiousness (*β* = −0.12, *p* < 0.05). Agreeableness did not exhibit any significant associations with emotional outcomes, suggesting that this trait may be less relevant in the context of music learning. These results indicate differential roles of personality traits in influencing emotional experiences during music learning.

Table 8 shows the variance in learning emotions explained by the Big Five personality traits. The model explained 42% of the variance in positive learning emotions, 38% of the variance in negative learning emotions, and 29% of the variance in boredom. According to Cohen’s criteria, these effect sizes can be interpreted as large. These R^2^ values suggest that personality traits are significant predictors of emotional experiences in music learning and account for substantial differences among students in their affective responses.

**Table 8:** Variance explained (R^2^) in outcome variables.

Outcome variable	R^2^	Effect size
Positive learning emotions	0.42	Large
Negative learning emotions	0.38	Large
Boredom	0.29	Medium-large

Effect size interpretation based on Cohen (1988): small (R^2^ = 0.02), medium (R^2^ = 0.13), large (R^2^ = 0.26).

The structural model explained substantial variance in learning emotions. Personality traits collectively accounted for 42% of the variance in positive learning emotions (R^2^ = 0.42), 38% of the variance in negative learning emotions (R^2^ = 0.38), and 29% of the variance in boredom (R^2^ = 0.29). These effect sizes can be characterized as large according to Cohen’s (1988) guidelines.

### 4.6 Summary of hypothesis testing

The hypothesized relationships between personality traits and learning emotions were largely supported. Extraversion (*β* = 0.35, *p* < 0.001) and openness (*β* = 0.28, *p* < 0.001) emerged as the strongest positive predictors of positive learning emotions, while neuroticism was the strongest predictor of negative learning emotions (*β* = 0.42, *p* < 0.001). Notably, openness demonstrated the strongest negative relationship with boredom (*β* = −0.32, *p* < 0.001), suggesting that music students high in openness to experience are particularly resistant to experiencing boredom during music learning activities. Agreeableness showed no significant relationships with any emotional outcomes, indicating that this trait may be less relevant in the music learning context.

**Figure 5:**
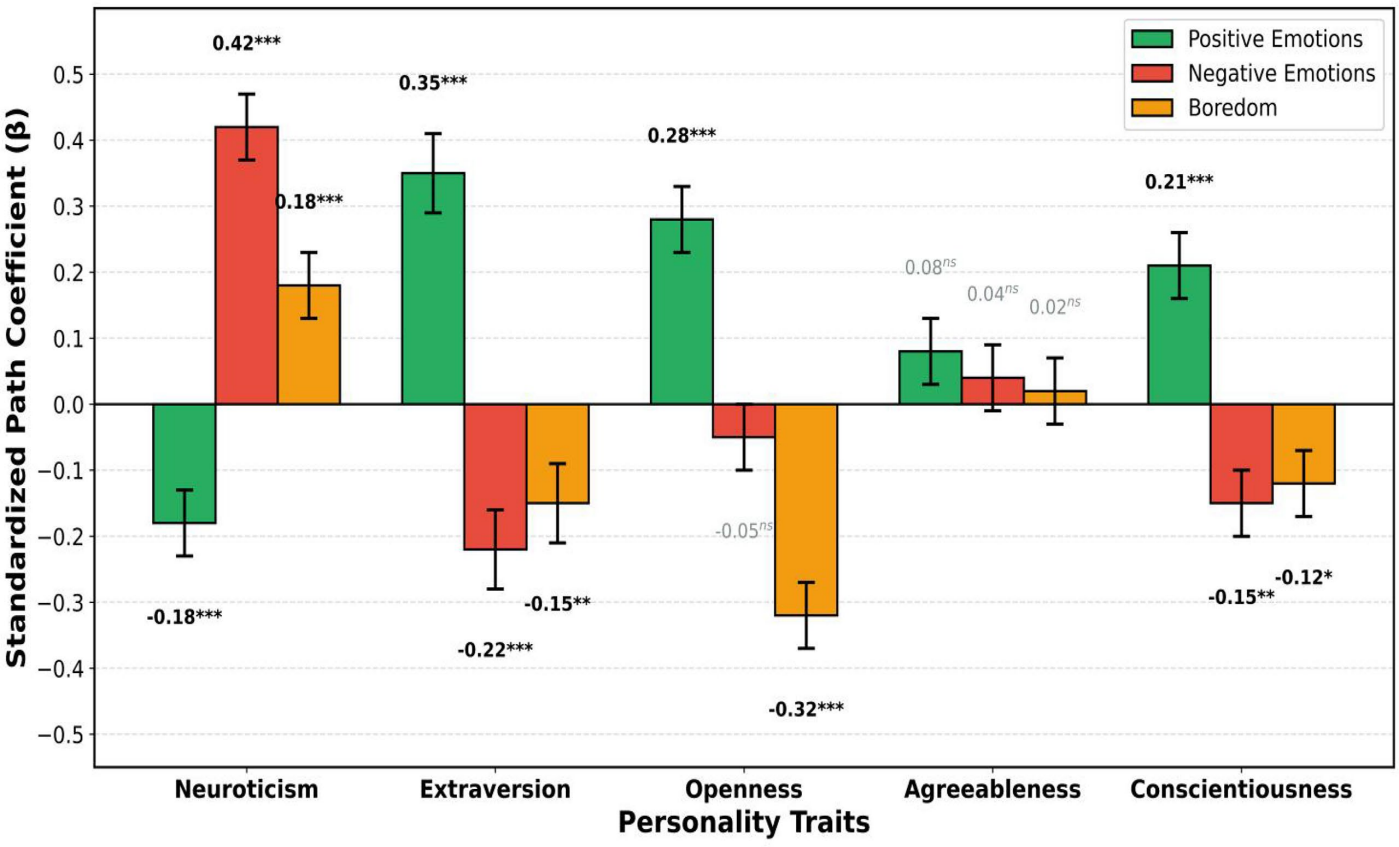
Comparative standardized path coefficients (β) for Big Five personality traits predicting three learning emotion outcomes. This grouped bar chart summarizes all 15 SEM path coefficients. Each cluster represents one personality trait (x-axis), with three bars per cluster: positive emotions (blue), negative emotions (orange), and boredom (green). Positive β values indicate higher trait scores predict higher outcome scores; negative β values indicate a protective/inverse relationship. Error bars represent standard errors of the path coefficients. Extraversion (*β* = 0.35) and openness (*β* = 0.28) were the strongest positive predictors of positive emotions; neuroticism was the dominant predictor of negative emotions (*β* = 0.42) and the strongest negative predictor of positive emotions (*β* = −0.18); openness demonstrated the strongest inverse relationship with boredom (*β* = −0.32). All agreeableness paths were non-significant (ns). *p < 0.05, **p < 0.01, ***p < 0.001, ns = not significant.

A comprehensive comparison of the standardized path coefficients across all personality–emotion relationships is presented in Figure 5, which shows a grouped bar chart comparing the standardized path coefficients (β) of Big Five personality traits predicting three learning emotion outcomes. Extraversion (*β* = 0.35) and openness (*β* = 0.28) exhibited the strongest positive relationships with positive emotions, whereas neuroticism showed the strongest positive association with negative emotions (*β* = 0.42) and the strongest negative association with positive emotions (*β* = −0.18). Openness demonstrated the strongest protective effect against boredom (*β* = −0.32), underscoring its relevance for sustained engagement in music learning. Conscientiousness was consistently associated with positive emotional outcomes across the three dimensions. Interestingly, agreeableness did not show any significant effects on emotional outcomes (all *p* > 0.05), as indicated by the gray-labeled non-significant coefficients. Error bars represent standard errors, indicating the precision of the estimated path coefficients.

## 5 Discussion

This study examines the relationship between Big Five personality traits and learning emotions among music students in Chinese undergraduate music students. Two primary objectives guided the investigation: (1) to evaluate the psychometric adequacy of instruments adapted for the music education context, and (2) to examine the predictive associations between stable personality dispositions and domain-specific learning emotional. The results indicate that personality traits explain substantial variance in learning emotions within creative and performance-based educational settings. These findings should be interpreted as associations rather than causal effects., these findings should be interpreted as evidence of association rather than causation, and their generalizability should be considered in light of the sample characteristics and cultural context. The findings offer theoretical and practical implications that warrant cautious, evidence-informed consideration.

### 5.1 Summary of findings

In this study, the relationships between the Big Five personality traits and learning emotions were analyzed in a sample of 298 Chinese music students, and the measurement instruments used in the music education context were validated. The measurement models of the Chinese Big Five Personality Inventory-Short Form (CBF-PI-SF) and the adapted Achievement Emotions Questionnaire-Short (AEQ-S) both demonstrated satisfactory fit, with Cronbach’s alpha values ranging from 0.77 to 0.87 and composite reliability scores ranging from 0.78 to 0.89. The hypothesized factor structures were supported by confirmatory factor analyses, with both the five-factor personality model (CFI = 0.912, TLI = 0.904, RMSEA = 0.051) and the eight-factor learning emotions model (CFI = 0.928, TLI = 0.918, RMSEA = 0.059) showing acceptable fit. Structural equation modeling results indicated that personality traits significantly predicted learning emotional outcomes. Extraversion emerged as the strongest positive predictor of positive emotions (*β* = 0.35, *p* < 0.001), followed by openness (*β* = 0.28, p < 0.001) and conscientiousness (*β* = 0.21, *p* < 0.001). Neuroticism was the strongest predictor of negative emotions (*β* = 0.42, *p* < 0.001), whereas openness showed the strongest protective effect against boredom (*β* = −0.32, *p* < 0.001). Agreeableness showed no significant associations with any emotional outcome. The model explained significant variance: 42, 38, and 29% in positive emotions, negative emotions, and boredom, respectively.

### 5.2 Personality traits as predictors of learning emotions

Consistent with the Control-Value Theory (Silvia et al., 2015) and existing evidence linking Big Five traits to affective outcomes in academic settings (Rodríguez Hernández et al., 2025), the present findings suggest that personality traits are meaningfully associated with learning emotions in music education. The finding that extraversion emerged as the strongest positive predictor of positive learning emotions is consistent with the well-established link between extraversion and positive affectivity (Rodríguez Hernández et al., 2025). Within the music learning context, this association may reflect heightened enjoyment during collaborative ensemble activities, greater pride following public performance, and a more optimistic orientation toward musical achievement. It should be noted, however, that these interpretations are inferential, as the present study did not directly measure the cognitive appraisal mechanisms proposed by CVT, nor did it distinguish between different learning activity types (e.g., solo practice vs. ensemble rehearsal). Future research employing experience-sampling methods or activity-specific assessments would be needed to substantiate these mechanistic explanations.

It is also notable that openness was closely associated with positive emotions and negatively associated with boredom, reflecting the creative nature of music education. Music learning may be particularly engaging for students high in openness, who tend to exhibit intellectual curiosity and aesthetic sensitivity. The diversity of music education provides ample opportunities for intellectual exploration and artistic engagement that align with the psychological tendencies of individuals high in openness.

The dominant predictive role of neuroticism for negative learning emotions is consistent with the trait’s well-documented association with negative affectivity and emotional instability (Rodríguez Hernández et al., 2025). The performance-evaluative and technically demanding nature of music education may constitute a particularly potent context for the expression of neurotic tendencies, potentially amplifying experiences of anxiety, shame, and hopelessness in students with elevated neuroticism scores. These findings tentatively suggest the value of targeted emotional regulation support for high-neuroticism music students; however, causal attribution cannot be established from the present cross-sectional data, and individual-level prediction based on trait scores alone would be inappropriate without longitudinal validation.

Conscientiousness showed positive associations with positive emotions and negative associations with negative emotions and boredom. Greater positive emotions may be associated with satisfaction derived from systematic skill development and goal attainment among conscientious individuals. The non-significant findings for agreeableness suggest that individual music learning may be less influenced by interpersonal dispositions, indicating domain-specific boundary conditions of personality–emotion interactions.

### 5.3 Theoretical implications

The current results contribute to theoretical knowledge in several respects. To begin with, the study applies control-value theory to the music education field, implying that personality traits, as distal antecedents of achievement emotions, operate through their effects on control and value appraisals. The substantial variance explained (R^2^ = 0.29 to 0.42) indicates that music learning emotional experiences are also significantly influenced by stable personality dispositions.

Second, the pattern of differences across emotion types supports the dimensional conceptualization of academic emotions. The finding that openness was the only predictor of boredom suggests that this emotion may function differently in aesthetic learning settings and may be particularly sensitive to individual differences in intellectual interest and aesthetic sensitivity.

Third, the non-significant associations for agreeableness provide insight into the boundary conditions of personality–emotion relationships. Music learning may represent a domain in which interpersonal personality dimensions are less predictive than traits related to emotionality, reward sensitivity, intellectual engagement, and goal-directed behavior.

Fourth, the effective validation of the adapted instruments contributes to ongoing debates on measurement specificity and generality, suggesting that while basic psychological constructs are consistent across domains, their specific expressions may vary.

### 5.4 Practical implications

The present findings have several practical implications for music educators and educational psychologists, although they should be interpreted cautiously until replicated in more diverse samples and contexts. Personality-informed awareness may help music educators recognize potential emotional vulnerabilities among students. In particular, students with high levels of neuroticism may benefit from proactive emotional regulation support, including evidence-based anxiety management strategies, graduated exposure to performance situations, and structured opportunities to develop coping skills. However, trait-based categorization also carries risks of stigmatization and deterministic interpretation. Personality profiles should therefore not be used as fixed diagnostic or tracking tools, but rather as a basis for flexible and individualized pedagogical support.

Second, music teachers may consider personality-based instructional differentiation. For students high in openness, activities with high levels of intellectual stimulation may enhance engagement and reduce boredom. For students lower in openness, more structured approaches with gradual exposure to novelty may be effective. Extraverted students may benefit from collaborative activities, whereas introverted students may benefit from opportunities for individual practice.

Third, curriculum design may incorporate personality considerations to create balanced learning experiences. The structuring of performance opportunities may vary in formality to accommodate students across different levels of neuroticism, with assessments including both individual and collaborative elements.

Fourth, the validated measures provide psychometrically reliable tools for assessing personality traits and learning emotions in music education, enabling systematic investigation of these experiences and supporting evidence-based practice.

### 5.5 Limitations and future directions

Several methodological limitations of the present study must be acknowledged. First, the cross-sectional design precludes causal inference. The observed associations between personality traits and learning emotions are consistent with the hypothesized directional relationships, but bidirectional and recursive effects cannot be ruled out. Longitudinal and experimental designs are needed to establish temporal precedence and directional causality.

Second, reliance on self-report measures introduces the possibility of common method variance and social desirability bias. Future research should incorporate multi-method approaches, including physiological indicators (e.g., heart rate variability as an index of anxiety), behavioral observations, and ecological momentary assessment (experience sampling), to triangulate and validate self-reported emotional experiences in naturalistic music learning contexts. To support replication, several methodological details warrant explicit documentation in future studies: the specific administration sequence of instruments, the criteria used to exclude participants based on attention checks, the exact wording of items modified during AEQ-S adaptation, and the software specifications and estimation procedures used in AMOS. The present study provides these details where feasible, but future research should adopt preregistration and open-materials practices to facilitate independent replication.

Third, the study was restricted to Chinese university music students, which limits the generalizability of the findings. Cross-cultural validation studies are needed to examine whether the relationships between personality and emotions operate similarly across different cultural and educational contexts.

Fourth, the mediating mechanisms were not investigated. Future studies should examine cognitive appraisals, particularly control and value perceptions, as potential mediators between personality and learning emotions.

Fifth, the study did not distinguish between specific learning situations. Future research could assess context-specific emotions in activities such as individual practice, ensemble rehearsal, and live performance.

Sixth, potential moderating variables such as years of musical training, level of achievement, and teacher characteristics were not examined. Investigating these boundary conditions would enhance both theoretical precision and practical applicability.

## 6 Conclusion

This study provides evidence regarding the associations between Big Five personality traits and learning emotions among Chinese undergraduate music students and contributes to the psychometric literature by validating adapted instruments for the music education context. Psychometric analyses confirmed that both the CBF-PI-SF and the adapted AEQ-S demonstrated acceptable reliability and factorial validity in this sample, supporting their use in future music education research. Structural equation modeling indicated that personality traits collectively predicted meaningful proportions of variance in positive learning emotions (R^2^ = 0.42), negative learning emotions (R^2^ = 0.38), and boredom (R^2^ = 0.29). These findings should be interpreted as preliminary evidence within the constraints of a cross-sectional, self-report design limited to one cultural context.

The results contribute to theory in two ways by showing that extraversion and openness are strongly associated with positive emotions in the music education context, whereas neuroticism plays a significant role in predicting negative emotions and susceptibility to boredom. The non-significant findings for agreeableness suggest domain-specific boundary conditions that warrant further investigation. In practice, these findings support an evidence-based approach to personality-informed teaching strategies, enabling music educators to design differentiated learning environments that accommodate diverse personality profiles and promote emotional well-being.

## Data Availability

The raw data supporting the conclusions of this article will be made available by the authors, without undue reservation.

## Footnotes

1
www.wjx.cn
